# Evidences on Molecules Most Frequently Included in Canine and Feline Complementary Feed to Support Liver Function

**DOI:** 10.1155/2020/9185759

**Published:** 2020-05-09

**Authors:** Andrea Marchegiani, Alessandro Fruganti, Alessandra Gavazza, Sara Mangiaterra, Alessia Candellone, Eleonora Fusi, Giacomo Rossi, Matteo Cerquetella

**Affiliations:** ^1^School of Biosciences and Veterinary Medicine, University of Camerino, Via Circonvallazione 93/95-62024, Matelica (MC), Italy; ^2^Department of Veterinary Science, University of Turin, Largo Braccini 2-10095, Grugliasco (TO), Italy; ^3^Department of Veterinary Medicine, University of Milan, Via dell'Università 6-26900, Lodi (LO), Italy

## Abstract

Numerous complementary feeds to support liver function are commercially available for small animals. Aiming to furnish a scientific support for clinicians/nutritionists that necessitate a complementary feed to support liver function in dogs and cats, with the present paper, we analyzed scientific evidences supporting the use, for this purpose, of ingredients/additives such as artichoke (*Cynara scolymus*), curcumin, dandelion (*Taraxacum officinale*), milk thistle (*Silybum marianum*), phosphatidylcholine, and S-adenosylmethionine. Although sustained by significant results, our review found only few scientific papers, and albeit we believe that they represent a significant aid in handling hepatopathies, in the authors' opinion, this topic probably deserves, and would benefit of, further studies.

## 1. Introduction

Acute and chronic hepatobiliary diseases are quite commonly reported in both dogs and cats [1–3]. These conditions recognize many different causes, as well as numerous therapeutic options also depending on several aspects such as underlying cause, chronicity, species involved, and others. Therefore, treatments reported variably include anti-inflammatory molecules (e.g., prednisolone, ursodeoxycholic acid, and S-adenosylmethionine), immunosuppressants (e.g. azathioprine), antifibrotics (e.g. colchicine and D-penicillamine), antioxidants (e.g. ursodeoxycholic acid, S-adenosylmethionine, vitamin E, N-acetylcysteine, and silymarin), dietary management, and management of any possible complications [2–4].

Despite that, well-designed clinical studies, such as randomized, double-blind, controlled trial, with an appropriate cohort of patients or adequate dosages of aforementioned compounds, tested as single agents or in combination, are scarce in veterinary medicine. Moreover, the vast majority of molecules to which “hepatoprotective” properties are attributed are mainly included and marketed as complementary feed. These are also intended as nutraceuticals, where a nutraceutical has been defined as “a food or part of a food, such as a dietary supplement, that has a medical or health benefit, including the prevention and treatment of disease” (Interview De Felice, 2011) [5] or as functional foods [6]. Interestingly, an international agreement on their definition has not yet been reached [6].

In veterinary medicine, from the legislative point of view, Regulation (EC) No 767/2009 [7], amending European Parliament and Council Regulation (EC) No 1831/2003, is the one that regulates the placing on the market and use of feed to safeguard both animal and human health. The regulation also includes, among others, definitions for feed additive, complementary feed, and feed intended for particular nutritional purposes, provides principles for labelling and presentation, and refers to the Commission Regulation (EU) No 68/2013 on the catalogue of feed materials [8] for their composition. This last one and the European Union Register of Feed Additives [9], pursuant to Regulation (EC) No 1831/2003, are indeed the main sources from which the components of complementary feed claimed as supporting liver function shall derive.

The aim of the present paper is to provide, through a literature review of their effects, a scientific support for clinicians/nutritionists that necessitate of complementary feed to support liver function in dogs and cats.

## 2. Search Criteria

Considering the molecules most frequently included in canine and feline complementary feed to support liver function, we researched in PubMed-NCBI (https://www.ncbi.nlm.nih.gov/pubmed/-last accessed 05/11/2019) the following terms: artichoke cat; artichoke dog; curcumin cat; curcumin dog; *Cynara scolymus* cat; *Cynara scolymus* dog; dandelion cat; dandelion dog; milk thistle cat; milk thistle dog; phosphatidylcholine cat; phosphatidylcholine liver dog; S-adenosyl methionine cat; S-adenosyl methionine dog; silybin cat; silybin dog; *Silybum marianum* cat; *Silybum marianum* dog; silymarin cat; silymarin dog; *Taraxacum officinale* cat; *Taraxacum officinale* dog; *Turmeric* cat; *Turmeric* dog. Starting from 1970 arriving to today, we overall found 977 articles, but once when excluded articles present in more than one search, articles not related to dogs or cats and liver function, articles in languages other than English, and studies referred to bioavailability/pharmacokinetics referring to dogs as experimental animals, only 18 articles remained. Of these, 3 were reviews, 8 research papers, 4 case reports, and 3 studies on cell cultures (Table 1).

## 3. Results of Literature Review

### 3.1. Silymarin, Silybin, *Silybum marianum*, Milk Thistle

*Silybum marianum* (L.) Gaernt., also known as milk thistle (MT), is an annual/biennial plant native of Mediterranean area (*Asteraceae* family) [27]. Silymarin is the major active component of the MT extract. In particular, silymarin is a mixture of flavonolignans (silybin, isosilybin, silychristin, isosilychristin, and silydianin) and a flavonoid (taxifolin) [28]. Its potentially beneficial effects on human health have been reported for centuries being used to treat kidney, spleen, liver, and gallbladder diseases [27]. The immunomodulatory, anti‐inflammatory, regenerative, antifibrotic, antioxidant, choleretic, and hepatoprotective actions are the most interesting effects of silymarin [27]. Extensive literature on the safety and potentially beneficial activities of MT has been published [29–32]. However, despite its increasing popularity and the extensive literature, the efficacy of MT extracts in patients affected by liver diseases is not fully understood. High‐quality standardized trials, with special attention to dose and treatment timing, are required in order to confirm the efficacy outlined in previous studies.

In veterinary medicine, an interesting study has recently been performed on 8 dogs presenting hepatopathy, given a phytocomplex of *Silybum marianum* pure extract. After two months of administration, a reduction of plasma alanine aminotransferase (ALT)/glutamate-pyruvate transaminase (GPT) activity was obtained; remarkably, the study also included other groups of dogs, given other nutraceuticals, and the activity of this enzyme only decreased in the hepatopathy group. Furthermore, in these dogs, the significant increase of paraoxonase and the up regulation of mitochondrial *SOD2* suggest an antioxidant activity for this phytocomplex in dogs. Unfortunately, as also stated by the authors, although results are interesting and suggest a positive effect of *Silybum marianum* in hepatopathies, the inclusion of only eight dogs in the study limits is its clinical relevance [12]. Remarkable are also the results obtained by Skorupski et al. that investigated the effect of the combination S-adenosylmethionine and silybin-phosphatidylcholine complex to prevent hepatopathy in dogs receiving lomustine, as affected by different kinds of neoplasm. Additionally, supplemented dogs showed a lower increase of ALT/GPT, alkaline phosphatase, bilirubin, and aspartate aminotransferase than dogs treated with lomustine alone [13]. Other less recent experimental studies on silymarin and silibinin have been performed on dogs given *Amanita phalloides*/phalloidin [19]. In one case, the effect of intravenous silymarin (and other molecules) was tested in experimentally poisoned dogs, and this molecule showed to prevent/reduce the increase of alkaline phosphatase (AP), GPT, and aspartate aminotransferase (AST)/glutamic-oxalacetic transaminase (GOT), reduce the severity of clinical signs, and also exerted some positive effects on coagulation [18]. Finally, intravenous administration of silibinin in beagles receiving a lyophilysate of *Amanita Phalloides*, containing *α*-, *β*-, and *γ*-amanitin and phallotoxins likewise resulted in a reduced hepatoxicity (measured by ALT/GPT, AST/GOT, AP, bilirubin, and prothrombin time), reduced liver damage (histological assessment), and absence of mortality [17]. Very few studies have been performed on the topic of the present paper on cats. One of these reports the efficacy of silymarin in healthy cats given acetaminophen; in these animals, levels of ALT/GPT, AST/GOT, alkaline phosphatase, lactate dehydrogenase, methemoglobin, and bilirubin did not increase as it happened in cats given acetaminophen alone [14].

Among the few papers we found with our search criteria, there are also a couple of case reports, in which a combination of active principles was used, including silybin and S-adenosylmethionine: one regarding a cat and one a dog. The former was a young adult female cat presented with acute neurological signs, pyrexia, and increased ALT/GPT, AST/GOT, and bilirubin, diagnosed after a complete diagnostic work up with hepatitis caused by *Yersinia pseudotuberculosis*. Successful treatment included antimicrobials, combination of S-adenosylmethionine and silybin, and fluid therapy [23]. The latter was about an adult female Pug dog diagnosed with toxic hepatopathy, caused by microcystins present in a dietary supplement that was given to the dog. After a very articulated therapeutic protocol including S-adenosylmethionine and silybin, ursodeoxycholic acid, N-acetylcysteine, antimicrobials, dolasetron, metoclopramide, vitamins K and B, and fluids, the cat fully recovered from hepatopathy [25].

To assess the possible efficacy of nutraceuticals on liver diseases, studies on cell cultures have also been carried out on single molecules/complexes or on their combination, with not always concordant results. A recent study reports the effects of a pretreatment with the combination of S-adenosylmethionine and silybin on primary canine hepatocytes exposed to the proinflammatory cytokine IL-1*β*, and these molecules are hypothesized as a possible support to the liver function. Indeed, the presence of this combination was associated with reduced levels of PGE_2_, IL-8, and macrophage chemotactic protein-1 (proinflammatory molecules induced by IL-1*β* administration) as well as to higher glutathione (GSH) levels (reduction of oxidative stress) [20]. Similarly, a previous study by the same authors reported the efficacy of silybin and of a silybin-phosphatidylcholine complex in canine primary hepatocyte cultures exposed to IL-1*β*, as demonstrated by a significant reduction of proinflammatory markers (PGE_2_, IL-8, and monocyte chemotactic protein-1) and by a decreased NF-*κ*B nuclear translocation, for the silybin-phosphatidylcholine complex [22]. On the other hand, in a study on cultured canine hepatocytes, the exposition to silibinin and simultaneously to *α*-amanitin, a known liver toxin from amanitin containing mushrooms (e.g. *Amanita phalloides*), did not show protective effects on those cells, which are measured by hepatocytes viability and lactate dehydrogenase activity measurement [21].

### 3.2. S-Adenosylmethionine (SAMe)

S-Adenosylmethionine (SAMe or AdoMet or SAM) is the active form of methionine. During its metabolism, mainly in the liver, methionine is converted to SAMe by the enzyme methionine adenosyltransferase (MAT), using ATP as cosubstrate [33]. In particular, SAMe is a fundamental methyl donor in transmethylation reactions of phospholipids, mainly phosphatidylcholine, which is essential to maintain the structure and function of cell membranes [34]. Moreover, at hepatic level, SAMe restores glutathione reserve and reduces the liver damage [35, 36]. As described in the review of Guo et al. [37], the meta-analysis of studies in humans (based on published randomized controlled trials), pointed out that in the treatment of liver diseases, SAMe enhances hepatic function parameters (total bilirubin and aspartate transaminase and not alanine aminotransferase). It is a safe dietary supplement and could improve the liver function, but in human medicine, the use of ursodeoxycholic acid (UDCA) and neo-minophagen C (SNMC) was more effective than SAMe in certain chronic hepatic conditions [37].

Despite the few manuscripts included with our research, an interesting prospective placebo-controlled crossover study, involving overall 12 healthy dogs receiving prednisolone, has been performed to investigate possible protective effects of S-adenosylmethionine. Although the study did not evidence a protective effect on hepatocyte glycogen vacuolization with this supplementation, it remarkably showed that SAMe could reduce the oxidative stress likely caused by steroids, maintaining the total glutathione concentration in erythrocytes stable and increasing the concentration of this molecules in the liver, positively affecting the redox status in these two substrates [15]. Also in cats, our literature review found a study, in which eighteen healthy subjects were tested for SAMe in acetaminophen-induced hematologic and hepatocellular oxidative stress. In these patients, SAMe administration was associated to a faster decrease of Heinz body presence as well as to a possible protective effect on PCV, although in the absence of a statistical significance [16].

Additionally, also two case reports were found. One was a case of an adult female Shetland sheepdog intoxicated with acetaminophen, presenting different hematological abnormalities and successfully treated with the administration of SAMe plus intravenous fluids and vitamin B, famotidine, cefazoline, and a packed RBC transfusion [26]. The last one was an adult male Chihuahua presenting acute hepatic failure due to xylitol ingestion and receiving a complete supportive therapy including N-acetylcysteine and S-adenosylmethionine [24].

These are not the only studies involving SAMe, the other few papers (research and case reports) have previously been cited [13, 20, 23, 25].

### 3.3. Phosphatidylcholine

Phosphatidylcholine is a phospholipid that represent 40–50% of cellular membranes and 70–95% of phospholipids in lipoproteins, surfactants, and bile. It is necessary for the synthesis of acetylcholine, an important neurotransmitter, as well as for kidney glomerular function and for mitochondrial function as betaine [38]. Phosphatidylcholine is mainly present in the liver that represents the major site of the choline metabolism. The equilibrium between phosphatidylcholine/choline/betaine uptake and anabolism with phosphatidylcholine catabolism and secretion is fundamental, and any alterations in choline and phosphatidylcholine metabolism could have important repercussion on liver function [39].

### 3.4. *Cynara scolymus*, Artichoke

In *Cynara* genus, globe artichoke (*Cynara cardunculus var. scolymus L*.) is well known in traditional medicine due to its beneficial effects in treatment of diseases of the biliary and digestive tracts, as well as in the treatment of scurvy and anemia. Native to the Mediterranean Basin, artichoke is cultivated all over the world for edible and medicinal purposes. In fact, this healthy food contains fibers, inulin, minerals (potassium, sodium, and phosphorus), vitamin C, and also polyphenolic compounds. These bioactive components, present mainly in the leaves rather than in the artichoke heads, include mono‐ and dicaffeoylquinic acids (e.g., chlorogenic acid and cynarin) and flavonoids (e.g., luteolin, apigenin, and their glucosides and rutinosides) [40]. Antioxidative, hepatoprotective, bile expelling, and lipid-lowering effects have been associated with the artichoke leaf extract [40]. In particular, Salekzamani et al. [41] reviewed the *Cynara scolymus* antioxidant activities in human, animal, and *in vitro* studies. Only data obtained in *in vitro* studies supported the antioxidant activities of artichoke (leaves or heads extracts) in the prevention or reduction of the oxidative stress. The human trials, due to the limited numbers (only two) showed no change or slight increase in the antioxidant status. The meta‐analysis of animal studies (23, mainly rats) pointed out that the artichoke extract supplementation had beneficial effect on antioxidant balance (increase superoxide dismutase, catalase, GSH, and glutathione peroxidase levels and decrease malondialdehyde level in liver and plasma) in animals with induced liver disease compared with others [41].

### 3.5. Curcumin, Turmeric

Turmeric (*Curcuma longa*) is a perennial herb member of the ginger family (*Zingiberaceae*). It is well known for its uses as curry powder in Asian cuisine and yellow pigment in the food processing industry. From the powdered rhizome of turmeric, several curcuminoids have been extracted, but the most prominent is called curcumin [42]. Although still debated, traditionally, curcumin, that present a polyphenolic structure, has been used for its anticancer, antidiabetic, anti-inflammatory, antimicrobial, antioxidant, hepatoprotective, immunomodulatory, and renoprotective properties as reported in literature [42, 43].

### 3.6. *Taraxacum officinale*, Dandelion

Plants from the genus *Taraxacum*, known as dandelion, have long been applied in traditional medicine to treat the liver disorders and some other diseases. Widely distributed in the warmer temperate areas, present in fields, roadsides, and ruderal sites, *Taraxacum* is a member of the family *Asteraceae* [44]. It is a perennial weed, containing terpenoid and sterol (principally taraxacin and taraxacerin) compounds, equally distributed in the roots, leaves, and flowers. Other terpene/sterol molecules are represented by *β*-amirin, taraxasterol, and taraxerol, but also free sterols (sitosterin, stigmasterin, and phytosterin) similar to bile [45]. Rubber, resins, tannins, fatty acids, levulose, a galactose polyholoside, arabinose, caffeic acid, *p*-hydroxyphenylacetic acid, asparagine, tyrosine, carotenoids, phytosterol, flavonoids, amino acids, saponins, and inulin could be also extracted [46]. Moreover, *T. officinale* is rich in minerals such as iron, copper, and potassium, as well as vitamins B1, PP, and D, and it contains high concentrations of vitamins A and C than other vegetables [46]. In addition to analgesic, anti-allergic, anticarcinogenic, anti-inflammatory, antithrombotic, diuretic, hypoglycemic, and prebiotic activities, also hepatoprotective, antifibrotic, antioxidant, antisteatotic, and choleretic effects have been attributed to *T. officinale* [44, 45]. After the consumption of *Taraxacum*, no toxicity has been reported. So, the GRAS seals qualification by the Food and Drug Administration (FDA) and the approval of Council of Europe [47, 48] for *Taraxacum* to be used as food. Attention must be paid in case of administration of roots in presence of obstruction of the bile ducts, as it is contraindicated, and it can only be used under medical supervision in cases of gallstones [46]. Furthermore, although as reported in the paper published by Devaraj in 2016, extensive literature on dandelion indicated the ability of the several extracts to influence the liver function, and most of them have been performed *in vitro* and *in vivo* (laboratory animals) models; therefore, attention must be made in transferring results to humans [44, 45].

Unfortunately, except for phosphatidylcholine [13, 22], our literature review did not produce any result, with the set criteria and filters, regarding *Cynara scolymus*, artichoke; curcumin; turmeric; *Taraxacum officinale*, dandelion.

## 4. Discussion and Conclusion

It is very interesting to notice that although only few research papers regarding the use of complementary feed to support liver function in dogs and cats are present in the literature, many different products are commercially available. Even though these few manuscripts are very interesting, following our search criteria, their reduced number as well as the number of subjects included do not provide strong evidence both in dogs and, especially, in cats. Furthermore, it must be considered that 4 out of 18 articles we found were case reports (Table 1). These kinds of papers do not fully meet the aim of our study because of their nature, and only Wallace et al. reported the use of a single “liver supporting molecule” (SAMe) [26] and not combinations as in others.

Our research has limitations as it looked only for some molecules, the most frequently included in veterinary complementary feed to support the liver function in dogs and cats, and it consequently failed to meet, for example, some other stimulating research articles always regarding this topic, although in healthy animals (one was an abstract) [49, 50] or in form of review [51]. In one case, SAMe was administered to healthy cats resulting, among other encouraging effects, in a positive influence on hepatic redox balance [50].

In conclusion, despite the wide presence of commercially available complementary feed to support liver function in dogs and cats and their widespread usage, studies on that topic (on molecules previously discussed) and on such species are rather scarce, while on the other hand, numerous studies have been carried out both in humans and in laboratory animals. Complementary feeds may represent a help in managing liver diseases in pets, but additional research studies are needed to confirm or exclude the presence of beneficial effects in different pathological conditions, including studies concerning the titration of active ingredients.

## Figures and Tables

**Table 1:** Results of the literature review with the set of criteria and filters.

	Type of manuscript	Molecule/s^*∗*^	Dog/cat	References
1	Review	—	Dog	Vandeweerd et al., 2013 [10]
2		—	Dog/cat	Webster and Cooper, 2009 [11]
3		—	Dog	Honeckman, 2003 [2]
4	Research paper	*Silybum marianum*	Dog	Sgorlon et al., 2016 [12]
5		S-Adenosylmethionine and silybin-phosphatidylcholine complex	Dog	Skorupski et al., 2011 [13]
6		Silymarin	Cat	Avizeh et al., 2010 [14]
7		S-Adenosylmethionine	Dog	Center et al., 2005b [15]
8		S-Adenosylmethionine	Cat	Webb et al., 2003 [16]
9		Silibinin	Dog	Vogel et al., 1984 [17]
10		Silymarin	Dog	Floersheim et al., 1978 [18]
11		Silymarin	Dog (and other animals)	Desplaces et al., 1975 [19]
12	Study on cell cultures	S-Adenosylmethionine and silybin	Dog	Au et al., 2013 [20]
13		Silibinin	Dog	Magdalan et al., 2009 [21]
14		Silybin; silybin-phosphatidylcholine complex	Dog	Au et al., 2010 [22]
15	Case report	S-Adenosylmethionine and silybin	Cat	Thompson, 2019 [23]
16		S-Adenosylmethionine	Dog	Schmid and Hovda, 2016 [24]
17		S-Adenosylmethionine and silybin	Dog	Bautista et al., 2015 [25]
18		S-Adenosylmethionine	Dog	Wallace et al., 2002 [26]

^*∗*^Only those molecules satisfying our search criteria are reported in the table. Other molecules such as, for example, ursodeoxycholic acid and N-acetylcysteine, included in some manuscripts reported in the references section and listed above but that did not meet search criteria or were studied for different diseases/conditions, were excluded from the table.
